# Evidence for Increased 5α-Reductase Activity During Early Childhood in Daughters of Women With Polycystic Ovary Syndrome

**DOI:** 10.1210/jc.2015-3926

**Published:** 2016-03-18

**Authors:** Laura C. Torchen, Jan Idkowiak, Naomi R. Fogel, Donna M. O'Neil, Cedric H. L. Shackleton, Wiebke Arlt, Andrea Dunaif

**Affiliations:** Division of Endocrinology, Metabolism, and Molecular Medicine (A.D.), Feinberg School of Medicine, Northwestern University, Chicago, Illinois 60611; Division of Pediatric Endocrinology (L.C.T., N.R.F.), Ann & Robert H. Lurie Children's Hospital of Chicago, Feinberg School of Medicine, Northwestern University, Chicago, Illinois 60611; Institute of Metabolism and Systems Research (J.I., D.M.O., C.H.L.S., W.A.), University of Birmingham, Birmingham B15 2TT, UK; Centre for Endocrinology, Diabetes and Metabolism (J.I., W.A.), Birmingham Health Partners, Birmingham B15 2TT, UK

## Abstract

**Context:**

Polycystic ovary syndrome (PCOS) is a heritable, complex genetic disease. Animal models suggest that androgen exposure at critical developmental stages contributes to disease pathogenesis. We hypothesized that genetic variation resulting in increased androgen production produces the phenotypic features of PCOS by programming during critical developmental periods. Although we have not found evidence for increased in utero androgen levels in cord blood in the daughters of women with PCOS (PCOS-d), target tissue androgen production may be amplified by increased 5α-reductase activity analogous to findings in adult affected women. It is possible to noninvasively test this hypothesis by examining urinary steroid metabolites.

**Objective:**

We performed this study to investigate whether PCOS-d have altered androgen metabolism during early childhood.

**Design, Setting, and Participants:**

Twenty-one PCOS-d, 1–3 years old, and 36 control girls of comparable age were studied at an academic medical center.

**Main Outcome Measures:**

Urinary steroid metabolites were measured by gas chromatography/mass spectrometry. Twenty-four hour steroid excretion rates and precursor to product ratios suggestive of 5α-reductase and 11β-hydroxysteroid dehydrogenase activities were calculated.

**Results:**

Age did not differ but weight for length Z-scores were higher in PCOS-d compared to control girls (*P* = .02). PCOS-d had increased 5α-tetrahydrocortisol:tetrahydrocortisol ratios (*P* = .04), suggesting increased global 5α-reductase activity. There was no evidence for differences in 11β-hydroxysteroid dehydrogenase activity. Steroid metabolite excretion was not correlated with weight.

**Conclusions:**

Our findings suggest that differences in androgen metabolism are present in early childhood in PCOS-d. Increased 5α-reductase activity could contribute to the development of PCOS by amplifying target tissue androgen action.

## Abstract (precis)

We investigated for alterations in steroid hormone metabolism and found evidence for increased 5α-reductase activity in daughters of women with PCOS during early childhood. This altered metabolism could result in increased androgen effects during early critical developmental periods and contribute to the pathogenesis of PCOS.

Polycystic ovary syndrome (PCOS) is a common, highly heritable disorder with substantial reproductive and metabolic morbidities (1). Hyperandrogenemia is the cardinal reproductive phenotype in adult female first-degree relatives of women with PCOS (2). Further, hyperandrogenemia is present in the daughters (PCOS-d) of affected women (3). We have found increased circulating testosterone (T) levels in PCOS-d as young as age 8 years (4). Although a number of susceptibility loci for PCOS have been reproducibly mapped (5, 6), these loci account for less than 5% of the approximately 70% heritability of PCOS (7). Epigenetic mechanisms may account for some of this so-called “missing heritability” that has been observed in PCOS and other complex genetic traits (8). Phenocopies of PCOS can be produced in animal models by exposure to androgens during gestation (9, 10), neonatally (11), or peripubertally (12). Therefore, the PCOS phenotype may result from the interactions of genetic variation resulting in hyperandrogenemia and the epigenetic actions of these androgens in utero as well as during critical postnatal developmental windows.

The source of androgens could be increased glandular androgen production. However, at birth, most studies (13, 14) suggest that cord blood androgen levels in PCOS-d are decreased. It remains possible that target tissue androgen action is amplified by upregulation of 5α-reductase activity resulting in increased production of DHT from T (15). If such increases in 5α-reductase activity were the result of variation in PCOS susceptibility genes, target tissue androgen action would be enhanced in affected individuals when T was available.

Alterations in intracellular steroidogenic enzyme activity could also increase adrenal androgen production. Increased 5α-reductase or decreased 11β-hydroxysteroid dehydrogenase type 1 (11βHSD1) activity would enhance cortisol metabolism resulting in a compensatory increase in ACTH secretion and stimulation of adrenal steroidogenesis (16, 17). Indeed, evidence for increased 5α-reductase and decreased 11βHSD1 activity has been found in adult women with PCOS (16–21). It is possible to noninvasively assess global androgen metabolism by measuring urinary metabolites and precursor: product ratios. We tested the hypothesis that 5α-reductase activity is increased in PCOS-d, who are at genetically high risk for PCOS, by measuring urinary steroid metabolites.

## Materials and Methods

### Study population

Twenty-one PCOS-d and 36 control girls aged 1 to 3 years were studied. We chose this age range to ensure that all subjects were at a similar developmental stage when the mini-puberty of infancy was completed, but the infants were still in diapers for ease of urine collection (22). Target sample sizes of 20 cases and 40 controls were calculated for the primary end point, the ratio of 5α-reduced tetrahydrocortisol (5αTHF) to its 5β-reduced metabolite, THF (5αTHF/THF), a measure of 5α-reductase activity (17, 23), assuming 80% power and alpha of 0.05 based on the mean and SD of this ratio in previous studies comparing PCOS to control women (17, 23). Subjects were recruited by advertisements in local media and on-line, and by contacting women who have previously participated in our studies of PCOS and control adult women. PCOS-d had a mother who fulfilled National Institutes of Health criteria for PCOS (hyperandrogenism and oligo-anovulation with exclusion of other reproductive disorders (1) as confirmed before recruitment by us or by their personal physician. The mothers of control girls had regular menses every 27–35 days and no signs or symptoms of androgen excess by a validated questionnaire (2). All girls were in good health and not taking any medications known to alter steroid hormone metabolism. The Institutional Review Boards of the Feinberg School of Medicine, Northwestern University, and Ann & Robert H. Lurie Children's Hospital of Chicago approved this study. Written informed consent was obtained from the parent or legal guardian of all subjects.

### Data collection

Age, height, weight, medical and reproductive histories were ascertained by validated questionnaires (2) in PCOS and control mothers. Timed urine samples were obtained in PCOS-d and control girls through collection of consecutive wet diapers for a minimum of 10 and maximum of 24 hours. Pediatric growth records for height and weight were available in 21 PCOS-d and 30 control girls. Weight for length Z score was calculated based on the US Centers for Disease Control and Prevention 2000 growth charts (24).

### Assays

Urine was extracted from diapers as described previously (25) and urinary steroid metabolites measured by a quantitative gas chromatography-mass spectrometry (GC-MS) selected ion-monitoring method (18). Metabolite concentrations were corrected to reflect 24-hour excretion rates by applying the following formula: [(metabolite concentration (μg/L) × urine volume (L))/(24/urine collection time (h))]. Substrate-to-product metabolite ratios were calculated to reflect the in vivo net activity of steroidogenic enzymes. Specifically, the ratio of the glucocorticoid metabolite 5αTHF to its 5β-reduced metabolite, THF (5αTHF/THF), was the primary ratio used to assess 5α-reductase activity because of the low concentrations of androgen and mineralocorticoid metabolites in this age group (Table 2) (26). The following ratios were used to assess 11β-hydroxysteroid dehydrogenase type 1 (11β-HSD1) activity: (5αTHF + THF)/tetrahydrocortisone (THE), and cortols/cortolones (27). Total cortisol production was quantified by the sum of cortisol metabolites: THF + 5αTHF + cortisol + cortols (26). Total cortisone production was quantified by the sum cortisone metabolites: THE + cortisone + cortolones (26). Total glucocorticoid production was quantified by the sum of total cortisol and total cortisone production (26). Total androgen production was quantified by the sum of androsterone (An) + etiocholanolone (Et) (26).

**Table 1.** Baseline Characteristics

Mothers	PCOS Mothers n = 21	Control Mothers n = 36	*P* Value
Age, y	32 ± 4	33 ± 3	.77
BMI, kg/m^2^	31 ± 8	26 ± 7	.01

Daughters	PCOS-d n = 21	Control Girls n = 36	
Age, y	1.7 ± 0.6	1.9 ± 0.5	0.45
Weight for length Z-score^a^	0.43 ± 1.04	−0.29 ± 0.99	.02
Race/ethnicity^b^	86% White, non-Hispanic, 6% Hispanic, 6% Asian Indian, 3% Asian	76% White, non-Hispanic, 10% Hispanic, 10% Black, non-Hispanic, 5% Asian Indian	.33
Length of urine collection, h	17.8 ± 4.5	16.5 ± 4.7	.28

Data are presented as mean ± sd with the exception of the categorical variable of race/ethnicity, where the percentage of subjects included in each group are noted. *P* values listed from *t* tests unless noted otherwise.

a Weight and length data available on 21 PCOS-d and 30 control girls.

b Categorical variables analyzed by Fisher's exact test.

### Statistical analysis

Data were log-transformed when necessary to achieve homogeneity of variance. Differences between groups were assessed by two-tailed unpaired *t* tests or Mann-Whitney tests, if homogeneity of variance was not achieved. Because weight for length Z score was higher in the PCOS-d, Pearson or Spearman correlation, depending on the normality of the data, was performed on all endpoints to determine whether there was a significant correlation with weight for length Z score. Because there was no correlation of weight for length Z score with any endpoint, no statistical adjustment for weight was necessary. Statistical analyses were performed using SAS 9.4 (SAS Institute, Inc.). Categorical variables were compared by Fisher's exact test. Differences were considered to be significant at *P* ≤ .05. Data are reported as the untransformed mean ± SD.

## Results

The PCOS mothers had increased body mass index (BMI) compared to control mothers but their age did not differ (Table 1). Age and ethnicity of the PCOS-d and control girls were similar by design (Table 1). Weight for length Z score (*P* = .02, Table 1) was higher in the PCOS-d. The length of urine collection did not differ between the groups (*P* = .28, Table 1).

**Table 2.** Urinary Metabolite Excretion, PCOS Daughters and Control Girls

	PCOS-d n = 21	Control Girls n = 36	*P* Value	*P* Value Correlation Weight-for-Length Z Score
Cortisol metabolites				
THF	44.7 ± 41.3	39.0 ± 30.7	.64	.50
5α-THF	115.2 ± 130.6	68.8 ± 48.0	.13	.40
α-cortol	11.5 ± 11.0	7.4 ± 5.5	.07	.82
β-cortol	24.7 ± 25.9	16.8 ± 14.5	.27	.44
Cortisol^a^	9.4 ± 10.2	6.8 ± 9.1	.14	.87
Cortisone metabolites				
THE	192.4 ± 200.6	165.5 ± 132.9	.69	.56
α-cortolone^b^	40.4 ± 32.6	34.0 ± 28.6	.64	.73
β-cortolone^b^	37.1 ± 30.2	32.9 ± 24.2	.80	.84
Cortisone	6.2 ± 5.2	5.9 ± 6.2	.51	.85
Androgen metabolites				
An	2.9 ± 3.1	2.5 ± 2.8	.42	.45
Et	1.6 ± 1.4	1.6 ± 1.7	.59	.64
Mineralocorticoid metabolites				
THDOC	1.7 ± 2.0	1.2 ± 2.0	.06	.47
5α-THDOC	2.3 ± 2.2	1.4 ± 1.7	.05	.69
THA	11.1 ± 15.0	8.0 ± 6.5	.49	.35
5α-THA	12.9 ± 16.3	7.3 ± 5.6	.08	.25
THB	7.8 ± 11.9	4.5 ± 4.2	.22	.37
5α-THB	45.6 ± 74.1	19.8 ± 16.7	.07	.25

All metabolite concentrations reported in μg/24 h. The *t* tests were performed for two-group comparisons and Spearman correlations for correlation with weight for length Z-scores unless noted otherwise.

Abbreviations: THA, tetrahydro-11-dehydrocorticosterone; THB, tetrahydrocorticosterone; THDOC, tetrahydrodeoxycorticosterone; THF, tetrahydrocortisol.

a One outlier PCOS-d cortisol result excluded (result >4 sd above mean).

b Nonparametric tests performed for α-cortolone and β-cortolone because of a failure to achieve homogeneity of variance.

**Figure 1.**
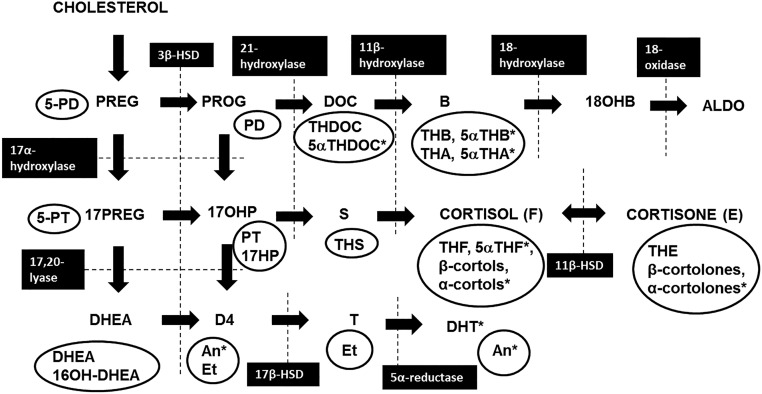
Steroidogenesis pathway highlighting steroidogenic enzyme activities and urinary steroid metabolites. Urinary steroid metabolites are circled and steroidogenic enzymes are indicated by black boxes. *The 5α-reduced metabolites. ALDO, aldosterone; B, corticosterone; 3β-HSD, 3β-hydroxysteroid dehydrogenase; 11β-HSD, 11β-hydroxysteroid dehydrogenase; 17β-HSD, 17β-hydroxysteroid dehydrogenase; D4, androstenedione; DOC, deoxycorticosterone; DHEA, dehydroepiandrosterone; 17HP, 17-OHpregnanolone; 18OHB, 18-hydrocxycorticosterone; 17OHP, 17-hydroxyprogesterone; PD, pregnanediol; 5PD, pregnenediol; PREG, pregnenolone; 17PREG, 17-hydroxypregnenolone; PROG, progesterone; PT, pregnanetriol; 5PT, 5-pregnenetriol; S, 11-deoxycortisol; THA, tetrahydro-11-dehydrocorticosterone; THB, tetrahydrocorticosterone; THDOC, tetrahydrodeoxycorticosterone; THF, tetrahydrocortisol; THS, tetrahydrodeoxycortisol.

**Figure 2.**
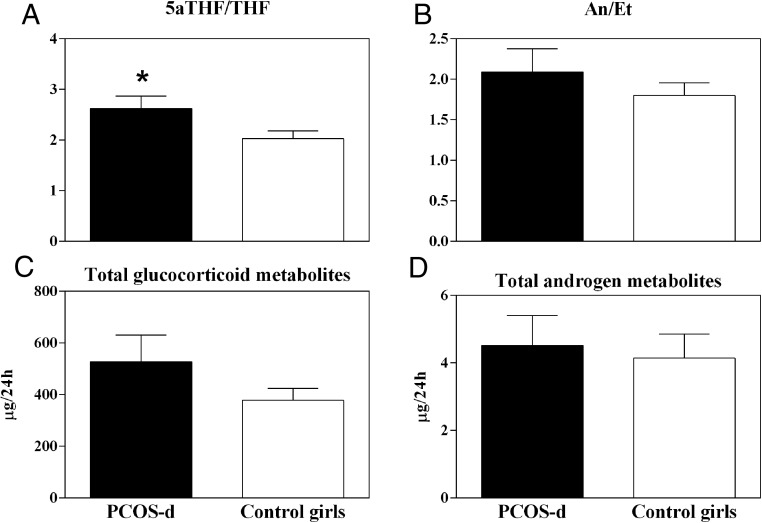
Metabolite ratios reflective of 5α-reductase activity and total cortisol and cortisone metabolite excretion. PCOS-d (black bars) had an increased 5αTHF/THF ratio compared to control girls (open bars), reflecting global increases in 5α-reductase activity (*P = .04, A). There was no difference in An/Et, a second ratio indicating 5α-reductase activity (*P* = .59, B). This discrepancy may be related to the very low concentrations of these androgen metabolites in this young cohort. There was no significant difference between the groups in total glucocorticoid metabolites (*P* = .30, C) or total androgen metabolites (*P* = .53, D). Data are mean and SEM.

Figure 1 depicts the steroidogenic enzyme and urinary steroid metabolites investigated. There were no differences between the groups in 24-hour excretion of any of the cortisol metabolites, although there was a trend toward increased α-cortol in PCOS-d (*P* = .07, Table 2). Excretion of the cortisone metabolites and androgen metabolites was also similar (Table 2). Excretion of the mineralocorticoid metabolites was similar between the groups, with the exception of 5α-tetrahydrodeoxycorticosterone, which was higher in the PCOS-d (*P* = .05, Table 2). Finally, excretion of total cortisol metabolites (211 ± 212 PCOS-d vs 139 ± 99 control girls, μg/24 hours, *P* = .16), total cortisone metabolites (276 ± 264 PCOS-d vs 238 ± 188 control girls, μg/24 hours, *P* = .55), total glucocorticoid metabolites (498 ± 466 PCOS-d vs 377 ± 281 control girls, μg/24 hours, *P* = .30, Figure 2) and total androgen metabolites (4.5 ± 4.0 PCOS-d vs 4.1 ± 4.3 control girls, μg/24 hours, *P* = .53, Figure 2) did not differ between the groups.

PCOS-d had significantly higher in vivo 5α-reductase net activity compared to control girls based on the 5αTHF/THF ratio (2.6 ± 1.1 PCOS-d vs 2.0 ± 0.9 control girls, *P* = .04, Figure 2), the most reliable measure of 5α-reductase net activity in this age group. The An/Et ratio (2.1 ± 1.3 PCOS-d vs 1.8 ± 0.9 control girls, *P* = .59, Figure 2) and other ratios of 5α- to 5β-reduced metabolites (Supplemental Table 1) did not differ between the two groups. The ratios reflective of 11β-HSD1 activity were similar between the groups: (5αTHF + THF)/THE (*P* = .17) and cortols/cortolones (*P* = .33). Weight for length Z score did not correlate with 5αTHF/THF, An/Et, (5αTHF + THF)/THE, or cortols/cortolones.

## Discussion

PCOS-d had evidence for increased global 5α-reductase activity analogous to findings in adult women with PCOS (16, 17, 19–21). 5α-reductase converts testosterone to its more potent 5α-reduced metabolite, DHT (15). Intracellular conversion of testosterone to DHT is responsible for androgen actions in most target tissues (15). In women, increased 5α-reductase activity has been associated with idiopathic hirsutism (28), androgenic alopecia (29), and acne (30). Increased 5α-reductase activity in specific tissues, such as the skin (31) and ovary (21), has been reported in women with PCOS. If genetic variation caused increased 5α-reductase activity in androgen target tissues of affected PCOS-d, it would result in the amplification of peripheral androgen action by increasing the intracellular production of DHT from T (32, 33). These findings are consistent with our hypothesis that increases in target tissue androgen production play a role in the development of PCOS.

Increased 5α-reductase activity would only be physiologically relevant when T was available. Although persistent increases in androgen secretion do not occur until the time of adrenarche (32), there are earlier developmental phases in which androgen secretion may be increased in girls. The fetal adrenal secretes DHEA, an androgen precursor that may undergo extraglandular metabolism to T (34). Although less is known about fetal ovarian androgen production, CYP17A1 is strongly expressed in fetal theca cells starting as early as 33 weeks, suggesting that intrauterine ovarian androgen production is possible (35). A “mini-puberty” of infancy occurs during the first 3–4 months of life during which time the hypothalamic-pituitary-gonadal axis is active, so it is biologically plausible that ovarian androgen production could be increased during this time (22). Following the first year of life, adrenal and ovarian androgen production remains quiescent until the time of adrenarche and subsequent puberty (32, 33). Detailed investigation of gonadal and adrenal steroid physiology during infancy and early childhood has been limited because of ethical and practical challenges of performing invasive studies in this age group (36). Analysis of urinary steroid metabolites provides a valuable means to noninvasively investigate sex steroid production in young children (18).

Androgen exposure in utero (9, 10), neonatally (11), or peripubertally (12) produces phenocopies of PCOS in several animal species (37). During gestation, robust placental aromatase activity likely protects the fetus from exposure to the increased circulating androgen levels in mothers with PCOS (38). Nevertheless, it remains possible that increases in fetal glandular or target tissue androgen production are present in females at risk for PCOS, such as those daughters of affected women who inherited PCOS susceptibility genes. No increase in cord blood androgen levels has been found in PCOS-d in studies using sensitive and specific testosterone assay methods (13, 14). However, increases in androgen production earlier in gestation or enhanced target tissue 5α-reductase activity resulting in local DHT increases could have escaped detection in studies using cord blood (13, 14). Alternatively, there could be increases in androgen production postnatally during other developmental windows, such as the “mini-puberty” of infancy (22).

In contrast to adult affected women, only the 5αTHF/THF ratio was increased in PCOS-d, whereas the An/Et ratio did not differ between the groups. However, the excretion of An and Et was near the limits of detection of the assay, limiting the utility of this ratio as a measure of 5α-reductase activity in children of this age, in whom androgen generation is physiologically very low (32, 33). In adult women with PCOS, 5α-reductase was positively correlated with body weight (17, 23). Although body weight was significantly higher in PCOS-d compared to control girls, there was no correlation of body weight with any urinary steroid metabolites suggesting weight differences did not contribute to the differences in 5α-reductase activity we found in PCOS-d.

Increased hepatic 5α-reductase activity may also enhance cortisol inactivation and clearance, resulting in a compensatory increase in ACTH secretion to normalize circulating cortisol levels, at the expense of increased adrenal androgen production (16, 17). The excretion of glucocorticoid metabolites was increased in adult women with PCOS (16, 17, 23, 39), consistent with ACTH-mediated cortisol production. Further, decreased activity of 11β-HSD1, which reduces inactive cortisone to cortisol, has been reported in some (23, 39) but not all (17) studies of adult affected women. This change may also result lower cortisol levels and in compensatory increases in ACTH release (23, 39). In the present study, neither glucocorticoid metabolite excretion nor 11β-HSD1 activity differed in PCOS-d compared to control girls. These findings suggest that increases in zona fasciculata activity are not important in the early origins of PCOS.

Our study has some potential limitations. First, urinary steroid metabolite ratios can estimate global enzyme activity, but do not reflect intracellular steroidogenic enzyme activity in target tissues (28–30). Nevertheless, this noninvasive approach is a valuable tool for investigation of alterations in steroid metabolism in infants and young children. Second, the difference we observed in 5αTHF/THF achieved only modest statistical significance (*P* = .04, Figure 2). However, this difference was similar in magnitude to differences in 5α-reductase activity observed in lean adult women with PCOS compared to lean control women (17, 23). Further, because genetic factors likely contributed to the alterations in steroidogenesis, these effects would be diluted because the PCOS-d group contained both genetically affected and unaffected girls.

In conclusion, our findings suggest that increased 5α-reductase activity is present in girls at risk for PCOS in early childhood. Hence, enzyme expression in target tissues may be primed toward androgen activation in genetically affected PCOS-d before the onset of increased glandular DHEA secretion during adrenarche (32, 40). These findings support our hypothesis that genetic variation leading to hyperandrogenemia, specifically increased target tissue production of DHT, contributes to the development of PCOS through epigenetic actions during critical developmental windows. Prospective cohort studies are needed to determine whether those PCOS-d with early childhood activation of 5α-reductase activity ultimately develop PCOS.
